# Case of Dr. W. G. Drake

**Published:** 1871-06

**Authors:** W. G. Drake

**Affiliations:** Atlanta


### CASE OF DR. W. G. DRAKE.

We publish below a letter from the above-named gentleman making application for membersnip in the Fulton County Medical Society. Dr. Drake's letter was submitted to a committee, which recommended his reception to membership *provided* Dr. Drake would *publish* his letter *for the information of the profession.* This he cheerfully csnsented to do, and therefore it appears in our Journal. Dr. Drake, by this action, has done what every honorable physician must commend and applaud, and furnishes the best evidence of his determination to conform his professional acts in harmony with the code. We commend his example to others:

ATLANTA, APRIL 18, 1871.

*To the President and Gentlemen of the Fulton county Medical Society:*

GENTLEMEN: I hereby make application for membership in the Fulton County Medical Society. In doing so, it is my duty to state—knowing your strict adhesion to ethical law—that in coming to your city to engage in practice, I announced myself as a specialist. This I very much regret now, as it was a violation of the Code, of which fact I was not aware when I so announced myself. I cheerfully agree to make any reparation in my power, and if admitted to membership in your Society, will unhesitatingly pledge myself to conform in every partiuclar to the code of medical ethics, and to the Constitution and By-Laws of your Society.

Gentlemen, I have the honor to be,
Your very obd't. serv't., W. G. DRAKE, M. D.

